# A Contemporary View of Natriuretic Peptides in the SARS-CoV-2 Era

**DOI:** 10.3389/fphys.2021.643721

**Published:** 2021-07-16

**Authors:** Speranza Rubattu, Giovanna Gallo, Massimo Volpe

**Affiliations:** ^1^Cardiology Unit, Department of Clinical and Molecular Medicine, Sapienza University of Rome, Sant’Andrea Hospital, Rome, Italy; ^2^IRCCS Neuromed, Pozzilli, Italy

**Keywords:** natriuretic peptides, RAAS, ACE2, Ang (1-7), COVID-19, ARNi

## Abstract

The heart releases natriuretic peptides (NPs) which represent an important hormonal axis with cardiorenal protective effects. In view of their properties, NPs have pathophysiologic, diagnostic and prognostic implications in several cardiovascular diseases (CVDs). Severe pulmonary inflammation, as induced by the SARS-COV2, may increase pulmonary pressure with potential influence on NPs release, whereby normal cardiovascular integrity becomes impaired. Moreover, pre-existing CVDs are strong negative prognostic factors since they exacerbate the effects of the viral infection and lead to worse outcomes. In this context, it may be expected that NPs exert a key protective role toward the virus infection whereas an impairment of NPs release contributes to the virus deleterious effects. In this review article we explore the potential involvement of NPs in the COVID-19 disease. To this aim, we will first focus on the interactions between NPs and the Ang II/ATIR arm of the renin-angiotensin-aldosterone system (RAAS) as well as with the protective ACE2/Ang (1-7) arm of the RAAS. Subsequently, we will review evidence that strongly supports the role of increased NT-proBNP level as a marker of cardiac damage and of worse prognosis in the COVID-19 affected patients. Finally, we will discuss the potential therapeutic benefits of these protective hormones toward the viral infection through their endothelial protective function, anti-inflammatory and anti-thrombotic effects. In conclusion, the potential implications of NPs in the SARS-CoV-2 infection, as discussed in our article, represent an important issue that deserves to be fully investigated.

## Introduction

The heart releases natriuretic peptides (NPs), including atrial and brain natriuretic peptides (ANP and BNP) (Woodard and Rosado, 2008; Volpe et al., 2014; Forte et al., 2019; Rubattu and Volpe, 2019), which play important functional effects within the heart, blood vessels and kidneys in order to maintain sodium electrolytes and blood pressure homeostasis (Potter et al., 2009). Within the heart, the myocytes stress is the main stimulus to release ANP from the atria and BNP from the ventricles. The endocrine role of the heart is associated to a local synthesis of NPs in several tissues, including the myocardium and the endothelium, and in few organs, including the brain and the kidneys, with the final goal to achieve a fine control of the mechanisms implicated in the cardiovascular functions (Atlas et al., 1986; Baron et al., 1989; Barbato et al., 2011; Arcari et al., 2020).

Of note, the COVID-19 disease selectively impacts the lungs with potential influence on the right ventricle due to potential increase in pulmonary pressure. As a consequence of the increased atrial distension, secondary to the increased pulmonary pressure, the release of ANP might also be affected.

Noteworthy, NPs play tight interactions with other hormonal systems within the cardiovascular apparatus. The most important is the renin-angiotensin-aldosterone system (RAAS), which has been implicated in the SARS-CoV-2 infection. In fact, ACE2 plays a role as the virus receptor (Gheblawi et al., 2020). We know that NPs inhibit renin and aldosterone synthesis in the kidneys and adrenal glands (Atlas et al., 1986; Cody et al., 1986). Furthermore, NPs counteract the salt-retaining, vasoconstrictive, pro-inflammatory, pro-hypertrophic and pro-fibrotic functions of the angiotensin converting enzyme 1 (ACE1)-Ang II-Ang II/type 1 receptor (AT1R) arm of the RAAS (Nishikimi et al., 2006). On the other hand, it is not surprising to observe that the protective arm of the RAAS, represented by ACE2, Angiotensin (1-7) (Ang 1-7) and Mas receptor, has a tight functional connection with NPs (Shah et al., 2010).

Based on the above-mentioned functional properties within the cardiovascular system and on their tight interactions with the RAAS, NPs may be involved in the SARS-CoV-2 infection.

The potential link between NPs and Covid19 will be the topic of the present review article. In particular, we will discuss all available evidence supporting the implications of NPs in the SARS-CoV-2 disease, involving their potential pathogenetic, prognostic and therapeutic roles.

## Interaction Between NPs and ACE1-Ang II-AT1R

**FIGURE 1:**
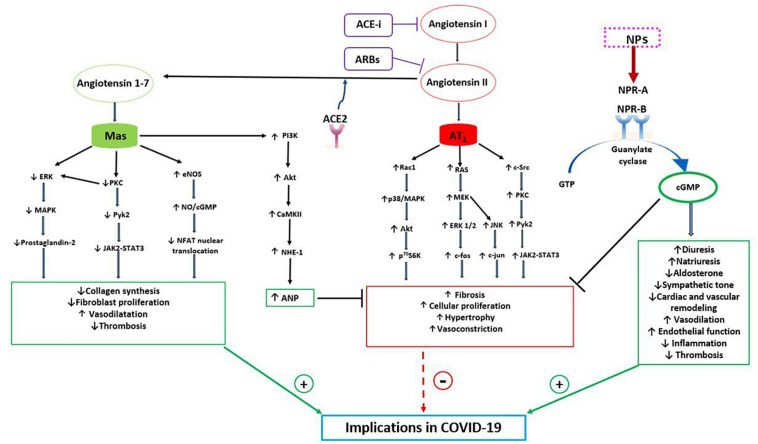
Bidirectional interaction between NPs and RAAS with potential implications in COVID-19. The RAAS may exert both protective and detrimental effects. Angiotensin II, through its receptor AT1, activates signaling pathways involved in fibrosis, cellular proliferation, hypertrophy and vasoconstriction. Indeed, AT1 stimulates the RAC1/p38/MAPK pathway which is involved in the activation of Akt, mTOR and its downstream target p^70^S6K. Moreover, AT1 activates both Ras/MEK/ERK pathway, which leads to an increased expression of c-fos mRNA, and JNK, which increases cJUN activity. AT1 also induces c-Src which, through PKC and PyK2, a non-receptor tyrosine kinase, stimulates JAK2-STAT3 signaling pathway. On the other hand, the protective arm of the RAAS is driven by ACE2-Ang (1-7)-Mas receptor, which plays complementary functions to the Angiotensin II-AT1 receptor. Mas receptor, indeed, reduces the activation of the MAPK, ERK and JAK2-STAT3 signaling pathways and increases the eNOS/NO pathway, down-regulating the NFAT transcription factor activity. As a consequence, Mas receptor activation reduces collagen synthesis, fibroblast activation and thrombosis. Moreover, Mas receptor activates PI3K/AKT and consequently CAMKII and NHE-1, increasing ANP gene expression, which further contributes to the protective role of ACE2-Ang (1-7). NPs indeed counteract the salt-retaining, vasoconstrictive, pro-inflammatory, pro-hypertrophic and pro-fibrotic functions of AT1. Through the stimulation of NPR-A and NPR-B and the production of cGMP, NPs increase diuresis, natriuresis, endothelial function and vasodilatation and reduce aldosterone production, sympathetic tone, inflammation and cardiac remodeling. As a consequence of their properties, NPs and Ang (1-7) may play a protective role during COVID-19 infection, reducing systemic inflammation, thrombosis, myocardial, lung and renal injury and improving prognosis. CAMKII, Ca 2 + /calmodulin-dependent protein kinase II; eNOS, endothelial nitric oxide synthase; ERK, extracellular-signal-regulated kinase; JAK, Janus kinase; JNK, c-JUN N-terminal kinase; MAPK, p38 mitogen-activated protein kinase; MEK, mitogen-activated protein kinase; NFAT, nuclear factor of activated T cells; NHE-1, sodium-hydrogen antiporter 1; NO, nitric oxide; p^70^S6K, ribosomal protein S6 kinase beta-1; PIK3, phosphatidylinositol 3-kinase; PKC, protein kinase C; PyK2, proline-rich tyrosine kinase 2; Rac1, Ras-related C3 botulinum toxin substrate; STAT3, signal transducer and activator of transcription 3.

ANP is the NPs family component that has been mostly investigated in this regard since its discovery. It has been observed that ANP acts in different ways to counteract the actions of the classical RAAS, driven by ACE1-Ang II-AT1R, in order to maintain blood pressure and electrolytes homeostasis (Atlas et al., 1986; Cody et al., 1986). In fact, through the interaction with NPRA-cGMP, it exerts vasodilation, that is most pronounced in Ang II-preconstricted blood vessels. It inhibits both renin secretion by the iuxtaglomerular cells within the kidney and ang II-induced aldosterone secretion by the adrenal cortex. Furthermore, ANP opposes the sodium-retaining action of aldosterone through its natriuretic effects (Atlas et al., 1986; Cody et al., 1986) (Figure 1). Thus, the two hormonal axes play a complementary hemodynamic role in the regulation of sodium-volume and of blood pressure. NPs and RAAS also exert opposing actions at the local tissue/cellular level to control the cardiovascular remodeling process. Herein, NPs promote beneficial effects with anti-proliferative, anti-inflammatory, anti-fibrotic and anti-hypertrophic actions (Volpe et al., 2014). The opposite holds true for the Ang II-AT1R arm which acts through the signaling pathway of the calcium/inositol triphosphate (Li et al., 2006). Therefore, the cardiovascular remodeling results from the effects exerted by the two opposing systems (Figure 1).

The complementary regulation of cardiovascular functions by NPs and RAAS becomes more evident in pathological conditions at both cardiac and vascular level. It is likely that it may play a relevant role in conditions where an excess of Ang II stimulates vasoconstriction, inflammation, oxidative stress, blood clotting such as the infection by SARS-CoV-2.

## Interaction Between ACE2-Ang (1-7) and NPs

The protective arm of the RAAS is driven by ACE2-Ang (1-7)-Mas receptor and it plays complementary functions to the Ang II-AT1R arm (Santos et al., 2003). Whereas AT1R signals through the calcium inositol triphosphate pathway to produce its effects, the Ang (1-7) acts through the Mas receptor to mediate vasodilation, protect endothelial function, and to oppose hypertrophy, fibrosis and thrombosis (Figure 1) (Santos et al., 2003; Wang et al., 2016). Activation of the ACE2-Ang (1-7)-Mas receptor reduces inflammation and fibrogenesis in several diseases (Zhuo et al., 2013). Interestingly, from the beginning of the SARS-CoV-2 pandemic and of its related COVID-19 outbreak, ACE2 has attracted increasing attention since it has been shown as the cell receptor through which the virus enters into the cells (Korber et al., 2020). However, it has been largely demonstrated that ACE2 counterbalances the detrimental effects of Ang II, reducing inflammation, fibrosis, thrombosis, vasoconstriction and increased vascular permeability and, as a consequence, the progression of lung and systemic damage during COVID-19 disease (Battistoni and Volpe, 2020). In addition, it has been reported that the pharmacological blockade of the RAAS, that influences the ACE2 level, have no impact on the clinical course and outcome of the SARS-CoV-2 infection (Battistoni and Volpe, 2020; Danser et al., 2020; Vaduganathan et al., 2020; Volpe et al., 2020).

Of note, a bidirectional interaction between NPs and ACE2 has been described in several previous studies. *In vitro*, ANP reduced Ang II induced hypertrophy of cardiac myocytes through the inhibition of the mitogen-activated protein kinase/extracellular-signal-regulated kinase (MAPK/ERK) pathway. Moreover, ANP, through the stimulation of NPRA and the production of cGMP, which upregulates the MAP phosphatase MKP1, prevented the reduction of ACE2 mRNA mediated by Ang II or endothelin-1 and preserved cellular growth (Gallagher et al., 2008).

On the other hand, Ang (1-7), the product of ACE2, has been demonstrated to stimulate ANP secretion through the Mas receptor/phosphatidylinositol 3-kinase/protein kinase B (Mas/PI3K/Akt) pathway. These data were confirmed by performing *ex vivo* high atrial pacing (Shah et al., 2010) and by using a rat model of cardiac hypertrophy, with evidence of Ang (1-7)-mediated increase of ANP release. In the presence of inhibitors of Mas, PI3K, Akt, NOS, sodium-hydrogen antiporter 1 (NHE-1) and Ca 2 + /calmodulin-dependent protein kinase II (CAMKII), Ang (1-7) was unable to induce ANP secretion, thus confirming the involvement of these molecular pathways in its ability to regulate ANP (Shah et al., 2010).

Interestingly, it has been reported that the ACE2 deficient mouse model shows increased glomerular damage as a result of increased oxidative stress, proinflammatory and profibrotic changes. The administration of ACE2 in this model upregulated renal ANP expression, with a 3-fold increase within 10 days compared to controls. ANP gene expression increased even further when Ang II was added to AT1R blockers, probably as a consequence of a rise of local unbound Ang II then degraded to Ang (1-7) by ACE2. This study suggested that ACE2 may directly regulate renal ANP production, independently from volume expansion and pressure overload (Bernardi et al., 2012).

The interaction between ACE2 and NPs was confirmed in another study which investigated the influence of sacubitril/valsartan (S/V), the main molecule of the ARNi pharmacological class, on the expression of RAAS genes and proteins in an animal model (Zhao et al., 2019). It is known that the concomitant selective inhibition of NEP and AT1R upon ARNi administration produces an increase of NPs, particularly of ANP (Rubattu et al., 2018; Ibrahim et al., 2019), level and prevents the potential effect of an excess of Ang II. In the mentioned study, S/V significantly upregulated ACE2 mRNA expression, compared to valsartan alone, probably as a consequence of the ANP increase in addition to the AT1R blockade (Zhao et al., 2019).

The stimulation of NPs may reveal crucial in conditions characterized by a defect of ACE2 and excessive Ang II, such as that observed in the SARS-CoV-2 infection.

## Potential Protective Role of NPs in COVID-19

As previously mentioned, at the vascular level, NPs regulate cellular growth and proliferation, preserving endothelial function and integrity as well as vascular tone. On the other hand, they oppose inflammation and atherosclerosis progression (Volpe et al., 2014; Forte et al., 2019; Rubattu and Volpe, 2019). In addition, ANP opposes blood clotting. In fact, it reduces *in vitro* the plasminogen activator inhibitor 1 (PAI-1) expression, a known modulator of fibrinolysis and a promoter of blood clotting (Bouchie et al., 1998). Evidence of a significant inverse relationship between ANP and PAI-1 levels was also reported in humans (Barbato et al., 2011).

Besides their well-described systemic hemodynamic and autocrine/paracrine functions within the cardiovascular system, NPs play an important protective role in the lungs. Herein, ANP reduces the secretion of inflammatory mediators and of endothelial and leukocyte-derived soluble adhesion molecules in response to lipopolysaccharide (LPS) and tumor necrosis factor α (TNFα). As a consequence, ANP is able to reduce lung endothelial permeability caused by inflammation and oxidative stress, avoiding the development of acute respiratory distress syndrome and improving arterial oxygenation during mechanical ventilation (Baron et al., 1989; Mitaka et al., 1998). As a further support to their relevant anti-inflammatory action, NPs have been shown to inhibit LPS/ATP-induced interleukin-1β secretion and to regulate the nuclear factor NF-kB/ERK pathway, THP-1 monocytes, and all elements of the NLR Family Pyrin Domain Containing 3/Apoptosis-associated Speck-like protein containing a Caspase-recruitment domain (NALP3/ASC)/caspase-1 inflammasome cascade (Mezzasoma et al., 2017).

Natriuretic and diuretic effects may also limit pulmonary edema and kidney damage. Moreover, based on the above described effects on blood clotting, NPs can contribute to inhibit the coagulopathy associated with COVID-19.

Furthermore, these hormones exert a well-known cardioprotective action toward myocarditis and acute cardiac dysfunction that may develop during infections (Currie et al., 2020). SARS-CoV-2 can certainly infect the heart, although the exact mechanism of cardiac involvement in COVID-19 has not been clearly understood. Based on both experimental and clinical evidence, both direct and indirect infections can take place. ACE2 is the receptor known to favor virus entry into the cells. However, a recent study demonstrated that extracellular vesicles from lung epithelial cells overexpressing SARS-CoV-2 were able to enter directly human cardiomyocytes *in vitro*, transferring viral RNA fragments into these cells and promoting inflammation (Kwon et al., 2020). Of note, Tavazzi et al. recently described a case of acute cardiac injury directly linked to myocardial localization of SARS-CoV-2, demonstrating low-grade myocardial inflammation and viral particles in the myocardium at the endomyocardial biopsy (Tavazzi et al., 2020). The precise role of NPs at the heart level in the context of SARS-CoV-2 infection remains to be established. The only study investigating a potential link of NPs with SARS-CoV-2 within the heart was conducted by performing a single-cell RNA sequencing (scRNA-seq) in both normal and failing hearts (Ma et al., 2021). Herein, ACE2 was found to be expressed in cardiomyocytes, vascular endothelial cells, fibroblasts, smooth muscle cells and immune cells in normal hearts, and its expression further increased in several cell subsets of the failing hearts (Ma et al., 2021). Importantly, BNP and ANP expression was upregulated in the more vulnerable ACE2-positive cardiomyocytes of failing hearts, along with a subset of genes favoring the viral infection (Ma et al., 2021). Although the latter evidence did not establish the exact type of relationship between ACE2 and NPs expression toward the virus entry and infection of the heart, it further suggested that NPs and ACE2 are tightly linked and may play an important role in the SARS-CoV-2 disease of HF patients. In particular, based on our knowledge, we can interpret the observed rise of ANP and BNP within the more vulnerable ACE2-positive cardiomyocytes as a reaction to the acute myocardial injury and as a protective response toward the inflammatory insult.

In fact, according to the available evidence, it has been proposed that COVID-19 patients with a deficiency of the NPs system, particularly obese subjects and black people, may have an increased risk of developing severe complications (Currie et al., 2020). This hypothesis certainly warrants further investigation.

## Use of NPs as Prognostic Markers in COVID-19

The role of NPs as potential prognostic biomarkers during COVID-19 infection represents an interesting issue and has attracted increasing attention in the medical community.

The cardiac release of NPs in patients with pneumonia or systemic infections may be explained by several pathophysiological mechanisms, such as increased myocardial wall stress as a consequence of hypoxia-induced pulmonary hypertension, development of cardiac injury due to the activation of inflammatory system, oxidative stress, demand-supply mismatch or the direct virus invasion, occurrence of renal failure and reduced NPs clearance (Thomas-Rüddel et al., 2019).

Current available studies have shown that increased NT-proBNP level is associated with an adverse clinical course during COVID-19 disease.

In a retrospective analysis by Guo et al. conducted in 187 patients, NT-proBNP level increased significantly during hospitalization only in those patients who died, without significant dynamic changes among survivors, thus predicting mortality independently from sex, age, hypertension, coronary heart disease, chronic obstructive pulmonary disease, myoglobin, creatin kinase-MB, high sensitivity troponin-I, white blood cells count, lymphocytes count, C-reactive protein, and procalcitonin (Guo et al., 2019). These findings were confirmed by a meta-analysis including 13 observational studies and a total of 2248 patients, which showed that elevated NT-proBNP level on admission was associated with a worse prognosis (Sorrentino et al., 2020). Patients with high BNP level had more severe cardiac injury with elevated troponin I level, a higher incidence of respiratory failure and a significantly increased mortality rate. Moreover, markers of coagulative disturbance were positively correlated with BNP level (Sorrentino et al., 2020). Another study conducted on 111 patients confirmed that BNP level was increased in patients with in-hospital mortality and it significantly correlated with age and previous CVD, whereas increased troponin I level correlated with age, PaO2/FIO2 and D-dimer (Arcari et al., 2020).

Accordingly, NPs level may be used as an indicator of clinical severity for SARS-CoV-2 infection, suggesting a more accurate cardiac evaluation, to exclude a direct or indirect myocardial involvement, to support clinical judgement and to tailor medical therapy.

## Potential Therapeutic Implications of ARNi in COVID-19

As stated above, S/V, the available component of the novel ARNi class of drugs, combines the inhibition of AT1R and of NEP, the latter responsible of NPs degradation. In addition, NEP degrades other vasodilator peptides such as bradykinin, substance P, enkephalins and adrenomedullin. Therefore, the effect of ARNi depends on a complex neuro-hormonal modulation with potentially greater beneficial effects compared to the selective RAAS inhibition (Bayes-Genis et al., 2016; Singh et al., 2017).

With regard to the trend of the different NPs plasma level after the initiation of S/V, NT-proBNP level decreases, as a consequence of the improvement of cardiac function and hemodynamic status representing a useful biomarker of treatment response, BNP level slightly increases due to its relatively low affinity to NEP, whereas ANP level consistently and substantially increases both in humans and experimental models, mediating most of the benefits of NEP inhibition (Rubattu et al., 2018; Ibrahim et al., 2019).

Importantly, S/V has been demonstrated to inhibit the secretion of granulocyte colony stimulating factor (G-CSF), granulocyte-macrophage colony stimulating factor (GM-CSF) and macrophage chemoattractant protein-1 (MCP-1), responsible of the so-called cytokine storm and of adverse clinical course (D’Elia et al., 2017).

Due to all beneficial properties dependent from its mechanisms of action, S/V has been already proposed as an early therapeutic strategy in all COVID-19 patients with the aim to reduce the progression of the disease, the need for intensive treatment with ventilators and other major complications and mortality (Acanfora et al., 2020; Mohammed El Tabaa and Mohammed El Tabaa, 2020). However, it should be underlined that, although S/V is recognized as a cornerstone of the therapeutic management of HFrEF, due to the impressive benefits on cardiovascular death and HF hospitalization, recommendations for its use apart from this clinical subset do not exist (McMurray et al., 2014; Ponikowski et al., 2016; Yancy et al., 2017).

In fact, a more specific, targeted approach to test the expected beneficial role of S/V in COVID-19 patients would be, first of all, to retrospectively investigate existing registries of hospitalized COVID-19 patients to find out whether, among subjects affected by HFrEF, those treated with S/V presented a lower disease incidence, a better clinical course (particularly in terms of intensive care unit access, mechanical ventilation and death) and a better prognosis, compared to patients who received other medications, including ACEI/ARBs (Rubattu et al., 2020). This key issue should become the target of future investigation during the ongoing COVID-19 pandemic.

## Author Contributions

SR, GG, and MV substantially contributed to the conception and design, acquisition of data, or analysis and interpretation of data, drafted the article and approved the final version to be published. All authors contributed to the article and approved the submitted version.

## Conflict of Interest

The authors declare that the research was conducted in the absence of any commercial or financial relationships that could be construed as a potential conflict of interest.
